# End-of-Life Care in the Last Three Months before Death in Older Patients with Cancer in Belgium: A Large Retrospective Cohort Study Using Data Linkage

**DOI:** 10.3390/cancers15133349

**Published:** 2023-06-26

**Authors:** Victoria Depoorter, Katrijn Vanschoenbeek, Lore Decoster, Geert Silversmit, Philip R. Debruyne, Inge De Groof, Dominique Bron, Frank Cornélis, Sylvie Luce, Christian Focan, Vincent Verschaeve, Gwenaëlle Debugne, Christine Langenaeken, Heidi Van Den Bulck, Jean-Charles Goeminne, Wesley Teurfs, Guy Jerusalem, Dirk Schrijvers, Bénédicte Petit, Marika Rasschaert, Jean-Philippe Praet, Katherine Vandenborre, Harlinde De Schutter, Koen Milisen, Johan Flamaing, Cindy Kenis, Freija Verdoodt, Hans Wildiers

**Affiliations:** 1 Department of Oncology, KU Leuven, 3000 Leuven, Belgium; 2 Research Department, Belgian Cancer Registry, 1210 Brussels, Belgium; 3 Department of Medical Oncology, Oncologisch Centrum, Universitair Ziekenhuis Brussel, Vrije Universiteit Brussel, 1090 Brussels, Belgium; 4 Division of Medical Oncology, Kortrijk Cancer Centre, AZ Groeninge, 8500 Kortrijk, Belgium; 5 School of Life Sciences, Medical Technology Research Centre (MTRC), Anglia Ruskin University, Cambridge CB1 1PT, UK; 6 School of Nursing & Midwifery, University of Plymouth, Plymouth PL4 8AA, UK; 7 Department of Geriatric Medicine, Iridium Cancer Network Antwerp, Sint-Augustinus, 2610 Wilrijk, Belgium; 8 Department of Hematology, ULB—Institute Jules Bordet, 1070 Brussels, Belgium; 9 Department of Medical Oncology, Cliniques Universitaires Saint-Luc—UCLouvain, 1200 Brussels, Belgium; 10 Department Medical Oncology, University Hospital Erasme, Université Libre de Bruxelles ULB, 1000 Brussels, Belgium; 11 Department of Oncology, Groupe Santé CHC-Liège, Clinique CHC-MontLégia, 4000 Liège, Belgium; 12 Department of Medical Oncology, GHDC Grand Hôpital de Charleroi, 6000 Charleroi, Belgium; 13 Department of Geriatric Medicine, Centre Hospitalier de Mouscron, 7700 Mouscron, Belgium; 14 Department of Medical Oncology, AZ Klina, 2930 Brasschaat, Belgium; 15 Department of Medical Oncology, Imelda Hospital, 2820 Bonheiden, Belgium; 16 Department of Medical Oncology, CHU-UCL-Namur, 5530 Namur, Belgium; 17 Department Medical Oncology, ZNA Stuivenberg, 2060 Antwerp, Belgium; 18 Department of Medical Oncology, Centre Hospitalier Universitaire Sart Tilman, Liège University, 4000 Liège, Belgium; 19 Department of Medical Oncology, ZNA Middelheim, 2020 Antwerp, Belgium; 20 Department of Medical Oncology, Centre Hospitalier Jolimont, 7100 La Louvière, Belgium; 21 Department of Medical Oncology, University Hospital Antwerp, 2650 Edegem, Belgium; 22 Department of Geriatric Medicine, CHU St-Pierre, Free Universities Brussels, 1000 Brussels, Belgium; 23 Department of Medical Oncology, AZ Vesalius, 3700 Tongeren, Belgium; 24 Academic Centre for Nursing and Midwifery, Department of Public Health and Primary Care, KU Leuven, 3000 Leuven, Belgium; 25 Department of Geriatric Medicine, University Hospitals Leuven, 3000 Leuven, Belgium; 26 Gerontology and Geriatrics, Department of Public Health and Primary Care, KU Leuven, 3000 Leuven, Belgium; 27 Department of General Medical Oncology, University Hospitals Leuven, 3000 Leuven, Belgium

**Keywords:** geriatric oncology, population-based data, specialized palliative care, terminal healthcare utilization

## Abstract

**Simple Summary:**

The care older patients with cancer receive in Belgium in the last months of life is not well defined. This study aimed to describe healthcare use at the end of life and explore which factors are associated with palliative care. This study shows that older patients with cancer in Belgium have frequent hospital admissions and emergency department visits before death and that more than half of the patients die in the hospital. Furthermore, we demonstrated that patients with limitations in function and cognition at cancer diagnosis receive less palliative care. This study gives insights into the type of care older patients depend on before death and which older patients receive less palliative care. Ultimately, healthcare use in the end-of-life period should be optimized, and palliative care should be made equally available in older patients with cancer.

**Abstract:**

This study aims to describe end-of-life (EOL) care in older patients with cancer and investigate the association between geriatric assessment (GA) results and specialized palliative care (SPC) use. Older patients with a new cancer diagnosis (2009–2015) originally included in a previous multicentric study were selected if they died before the end of follow-up (2019). At the time of cancer diagnosis, patients underwent geriatric screening with Geriatric 8 (G8) followed by GA in case of a G8 score ≤14/17. These data were linked to the cancer registry and healthcare reimbursement data for follow-up. EOL care was assessed in the last three months before death, and associations were analyzed using logistic regression. A total of 3546 deceased older patients with cancer with a median age of 79 years at diagnosis were included. Breast, colon, and lung cancer were the most common diagnoses. In the last three months of life, 76.3% were hospitalized, 49.1% had an emergency department visit, and 43.5% received SPC. In total, 55.0% died in the hospital (38.5% in a non-palliative care unit and 16.4% in a palliative care unit). In multivariable analyses, functional and cognitive impairment at cancer diagnosis was associated with less SPC. Further research on optimizing EOL healthcare utilization and broadening access to SPC is needed.

## 1. Introduction

End-of-life (EOL) care is the healthcare provided at the time nearing the end of a patient’s life. EOL is a resource-intensive period typically including hospital care, emergency care and many contacts with healthcare professionals [1,2]. In the EOL period, a transition from life-prolonging care to more comfort-oriented care is needed, but this shift often occurs (too) late in the illness trajectory [3]. Consequently, care at the EOL can often be considered inappropriate and overly intense, especially in patients with cancer [4,5]. 

Specialized palliative care (SPC) is an essential element of EOL care and an approach to improving the quality of life of patients with life-threatening illnesses and their families. This is obtained through the prevention and relief of suffering using a patient-centred approach [6]. Growing evidence supports the timely integration of palliative care in the cancer care continuum as it avoids unnecessary acute care use, reduces symptoms, and can even improve survival [7,8,9]. Despite the indisputable benefits, palliative care is not accessible to all patients in need, even in high-income countries [10,11]. In Belgium, palliative care is not considered a separate speciality, but structures and services are created specifically for the multidisciplinary care of palliative patients (i.e., specialized palliative care). Specialized palliative care by nurses and multidisciplinary palliative support teams is available for in-home settings. In hospital settings, palliative beds are clustered in specialized palliative care units, and mobile palliative care teams are available to support patients hospitalized in non-palliative units. In nursing homes, specialized palliative care is also provided by mobile teams. General practitioners (GP) and geriatricians (among other doctors) are involved in and trained in basic palliative care, but levels of training differ [12].

Since cancer typically affects individuals at an older age, studying EOL care in this population is especially relevant from a societal and individual perspective [13]. Older patients require special attention throughout their cancer care trajectory from initial treatment to death because of coinciding health and age-related conditions [14]. Geriatric screening (GS) and geriatric assessment (GA), part of the Comprehensive Geriatric Assessment, are used to identify geriatric syndromes such as frailty, falls, and cognitive impairment and to guide geriatric interventions [15]. Performing GS and GA at cancer diagnosis can improve care through individualization of treatment decisions to avoid overtreatment and undertreatment, facilitating patient-physician communication, decreasing treatment-related toxicity, improving quality of life, and reducing hospitalizations [16,17,18,19].

Various studies, including studies from Belgium, have examined the nature and quality of EOL care in patients that died from cancer [4,20,21]. Other researchers have also shown that palliative care use decreases with age [5,22,23]. Nevertheless, only a few investigators specifically focus on EOL care in older patients diagnosed with cancer that died of any cause. Almost all these studies utilize population-based data, i.e., healthcare reimbursement and cancer registry data, which lack clinical GS and GA data. 

Through a unique linkage of clinical data from a previous observational study on GS and GA (GS/GA study) and population-based data, we will explore EOL care in older patients with cancer. Firstly, the aim is to describe EOL care (healthcare service use, medication use and SPC use) and circumstances of death (place and cause of death). Secondly, this study investigates the association between baseline clinical, oncological, sociodemographic, and GS/GA data from cancer diagnosis and SPC use in older patients. This way, we can explore which (age-related) factors are associated with less SPC use. This might give insights into which factors influence SPC access in older patients with cancer. 

## 2. Materials and Methods

### 2.1. Study Design

For the current study, patients aged 70 years and older with a new cancer diagnosis (registered date of diagnosis within six months before or two months after the date of inclusion) that were included in a previous GS/GA study [24,25,26] and if they had died of any cause before the end of follow-up (1 March 2019) were selected. The previous GS/GA study consisted of three consecutive multicentric prospective observational cohort studies (Study 1 October 2009–July 2011, *n* = 10 hospitals; Study 2 August 2011–July 2012, *n* = 9 hospitals; Study 3 November 2012–February 2015, *n* = 22 hospitals) and evaluated the implementation of GS/GA in Belgian patients with cancer. In total, 22 hospitals were involved in the GS/GA study. The hospitals are spread out over the three Belgian regions and include academic and non-academic hospitals.

For all patients, the GS/GA data were linked to population-based data, including registry data from the Belgian Cancer Registry (BCR) and healthcare reimbursement data from the InterMutualistic Agency (IMA) to evaluate EOL care. The study was approved by the Ethics Committee of all 22 hospitals of the GS/GA study and by the Belgian Information Security Committee. The data were linked deterministically, but researchers only had access to pseudonymized data. The linking procedure, data sources (including data quality) and patient selection have been described in detail previously [27].

### 2.2. Data Sources

From the GS/GA study (2009–2015), baseline patient, clinical, and sociodemographic variables (age, sex, Charlson Comorbidity Index [CCI] [28], Eastern Cooperative Oncology Group Performance Status [ECOG-PS] [29], polypharmacy, attained educational level, marital status) were derived. For the CCI, the tumor leading to inclusion in the study was excluded. Furthermore, Geriatric 8 (G8) results and GA results (in case of an abnormal G8 score [≤14/17]) were provided. 

From the BCR, cancer registry data (2009–2015) were obtained, including date of cancer diagnosis, tumor type (International Classification of Diseases, Tenth-Revision [ICD-10]) and stage (TNM classification 6th–7th edition for applicable periods) [30,31,32]. In addition, the date of death and causes of death of all patients were made available through BCR. The date of death was obtained from the Belgian Crossroads Bank for Social Security (available until 1 April 2020), and death certificates coded in ICD-10 from the regional authorities (‘Agentschap Zorg en Gezondheid’, ‘Observatoire de la Santé et du Social de Bruxelles-Capitale’, and ‘Agence pour une Vie de Qualité’ [AVIQ]; available until 31 December 2017). 

From IMA, healthcare reimbursement data were obtained that contained healthcare services use (billed medical acts, hospital stays, nursing home stays) and medication on prescription dispensed in hospital and/or public pharmacies (available until 1 March 2019). Healthcare services were identified based on charged national nomenclature codes (available online via ‘Nomensoft’ [33]) and medication based on Anatomical Therapeutic Chemical (ATC) classification codes [34]. 

### 2.3. Study Participants

The current study included older patients with a new cancer diagnosis if they had GS/GA, BCR and IMA data available. Analysis was restricted to patients who underwent GS/GA within two months before and up to six months after the new cancer diagnosis. To evaluate healthcare service and medication use in the last three months of life, analyses were limited to patients who died before the end of follow-up (1 March 2019) and who survived at least three months after the performance of GS/GA. This allowed a full 3-month observation period before death for each patient.

### 2.4. Geriatric Screening and Geriatric Assessment

GS was performed with the G8, the score ranges from 0–17, and a score ≤14 indicates an abnormal result. Only in case of an abnormal result a 7-item GA was performed. The GA consisted of the following scales: Katz’s Activities of Daily Living (ADL), Lawton’s Instrumental Activities of Daily Living (IADL), Number of falls in the past year, Visual Analogue Scale (VAS) for pain, Mini-Mental State Examination (MMSE), 4-item Geriatric Depression Scale (GDS-4), and Mini Nutritional Assessment-Short Form (MNA-SF). Cut-off values used for each scale are summarized in Supplementary Table S1. 

### 2.5. Outcomes

The main outcomes were (1) the percentage of deceased cancer patients using selected healthcare services and (2) the number of days using those services in the last three months before death. Healthcare services included inpatient hospital admissions, intensive care unit (ICU) admissions, emergency department (ED) visits, contacts with general practitioners (GP), contacts with specialists, home care by nurses, and nursing home (NH) admissions. More details on outcomes and corresponding Belgian nomenclature codes for reimbursement are available in Supplementary Tables S2 and S3. Healthcare service was calculated as the percentage of patients using the specific service in a specified period. In addition, in the case of usage, the number of days with contact or use in the last three months were counted, except for specialist contacts which were counted as the number of contacts (multiple specialist contacts on one day was possible). The percentage of patients with a prescription for a certain medication dispensed in the last three months before death was calculated for medication use. Systemic anti-neoplastic therapy (excluding endocrine therapy), anti-neoplastic endocrine therapy, systemic corticosteroids, opioids, and midazolam use for palliative sedation and/or symptom control were identified based on ATC codes (details in Supplementary Table S4). 

In addition, a composite outcome was created to describe the use of specialized palliative care (SPC). This indicated either a palliative care unit admission, use of palliative home care by a nurse and/or use of a multidisciplinary palliative support team at home three months before death. The percentage of patients and the number of days using SPC in the last three months before death were calculated. In addition, the median start before the death of each type of SPC was measured (first day with billed SPC in the last three months before death). Mobile palliative care teams coordinating specialized palliative care for patients in non-palliative units in hospitals and nursing homes were not included as they cannot be distinguished in IMA data. The same applies to basic palliative care doctors provide in non-palliative care units. Therefore, our results on SPC use do not apply to mobile palliative care teams in hospitals or nursing homes. This study’s limitation related to the use of healthcare reimbursement data is further explained in the discussion.

For the circumstances of death, the cause and place of death were analyzed. The cause of death was obtained from the underlying cause on the death certificate. ICD-10 C00-D48 were considered to indicate cancer-related deaths. Place of death was derived from IMA data based on healthcare service use on the date of death and one day before, prioritising hospital care over nursing home care. In case no billed activity for a hospital or nursing home was found, patients were considered to have died at home. 

### 2.6. Statistical Analysis

The characteristics of the cohort were described using frequencies for categorical variables and the median with interquartile range (IQR) for continuous variables. The baseline was defined as the date of performance of the GS/GA (date GS/GA). Dichotomous variables were generated for each GA domain based on cut-off scores as summarized in Supplementary Table S1. Outcomes were assessed in the last three months (=90 days) before death (day of death = d0, start point = d89 before death). 

For healthcare services, we calculated the proportion of patients with use or contact for at least one day during the last three months of life. The number of days with use or contact during the last three months of life was also calculated. In graphs, we displayed the proportion of patients with use or contact for at least one day per 10-day interval in the last three months. The proportion of patients with use or contact per day in the last ten days is available in Supplementary Materials (Figure S1). For medication use, we calculated the proportion of patients with at least one dispensed medication during the last three months of life. Medication use was not analyzed in shorter periods as medications are often prescribed for longer periods. 

To identify factors associated with SPC use (dichotomous variable), logistic regression was performed. First, to estimate unadjusted odds ratios with 95% confidence intervals (95% CI). Subsequently, multivariable logistic regression models were constructed to estimate odds ratios and 95% CI, adjusted for co-variates (the full model was fitted, with no selection of covariates). Adjustments were made for age (70–74, 75–79, 80–84, ≥85), sex (female, male), tumor type (22 tumor types and one category ‘other’ as displayed in Supplementary Table S5), stage (I, II, III, IV, not applicable [NA], unknown), CCI (no comorbidity: score 0; mild comorbidity: score 1–2; moderate comorbidity: score 3–4; severe comorbidity: score ≥5; missing), attained educational level (higher education, upper secondary education, lower secondary education, primary education, illiterate, other, missing), marital status (partnered, not partnered, other, missing), cohort identification (study 1, study 2, study 3) and time between GS/GA and death (4 categories based on quartiles). In a sensitivity analysis, baseline nursing home resident status (no, yes) was added as a covariate in the multivariable model, and results are displayed in Supplementary Materials.

A two-tailed *p*-value < 0.05 was considered statistically significant. Missing baseline information was assigned to a separate category. Only for the cause of death and in analyses with GA domains patients with missing data were omitted. All analyses were conducted in SAS version 9.4 (SAS Institute Inc., Nary, NC, USA).

## 3. Results

### 3.1. Patient Population and Baseline Characteristics

**Figure 1:**
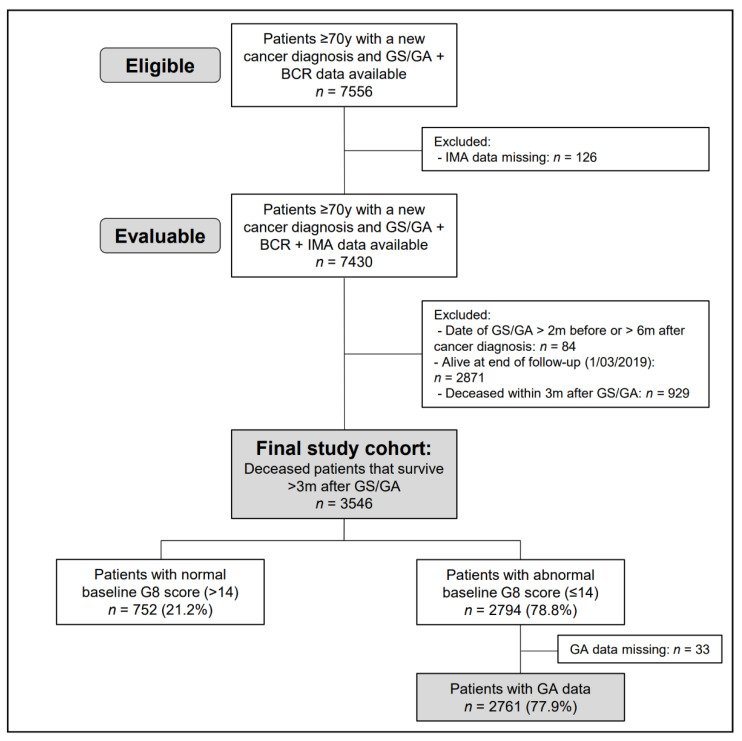
Patient Flow Chart. (Abbreviations: y, years; GS, geriatric screening; GA, geriatric assessment; BCR, Belgian Cancer Registry; IMA, InterMutualistic Agency; m, months; G8: Geriatric 8).

A total of 3546 deceased patients who survived at least three months after GS/GA were included in the current study (Figure 1). Patient characteristics are displayed in Table 1. The median age at baseline was 79 years (IQR: 75–83), and 53.6% of patients were female. The most common diagnoses were breast (18.5%), colon (15.1%), lung (14.5%), rectum (6.1%) and pancreas (4.3%) cancer. In 27.0%, the patients’ cancer was diagnosed at stage IV. According to CCI, most patients had mild comorbidity at baseline (40.8%). In 34.2% of patients, G8 geriatric screening was performed less than one year before death.

### 3.2. Geriatric Screening and Geriatric Assessment

GS and/or GA were performed at the date of inclusion which was a median of 17 days (IQR 7–34) after a cancer diagnosis. An abnormal baseline G8 score (≤14/17) occurred in 2794 patients (78.8%). A GA was available for 2761 (98.8%) of these patients, and baseline characteristics for this group are also displayed in Table 1. Most patients were at risk for malnutrition (82.2%) according to the MNA-SF scale, followed by at risk for functional dependence according to the IADL scale (67.0%) and ADL scale (58.1%). The IADL includes more complex tasks essential to living independently, such as managing medication, preparing meals, and managing finances, while the ADL includes more basic self-care tasks, such as bathing and dressing. A total of 57.8% were at risk for depression (GDS-4). Mild to severe pain (VAS) was reported by 49.4% of the patients. A fall history in the past year was present in 37.7% of the patients, and 21.7% had mild to severe cognitive impairment (MMSE).

### 3.3. Healthcare Services Use and Medication Use in EOL

In the last three months before death, 2705 (76.3%) patients had a hospital admission and in total a median of 20 days (IQR 9–36) were spent in the hospital (Table 2). A total of 371 (10.5%) patients spent at least one day in ICU with a median of 4 days (IQR 2–8), and 1743 (49.1%) patients had at least one ED visit with a median of 1 visit (IQR 1–2). In total, 3148 (89.9%) patients had at least one contact with a GP with a median of 6 contacts (IQR 3–10), and 2147 (60.5%) patients had at least one contact with a specialist with a median of 2 contacts (IQR 1–3). Normal home care was received by 1895 (53.4%) patients with a median of 26 days (IQR 8–58), and 856 (24.1%) patients spent at least one day in a nursing home with a median of 83 days (IQR 53–90). 

**Figure 2:**
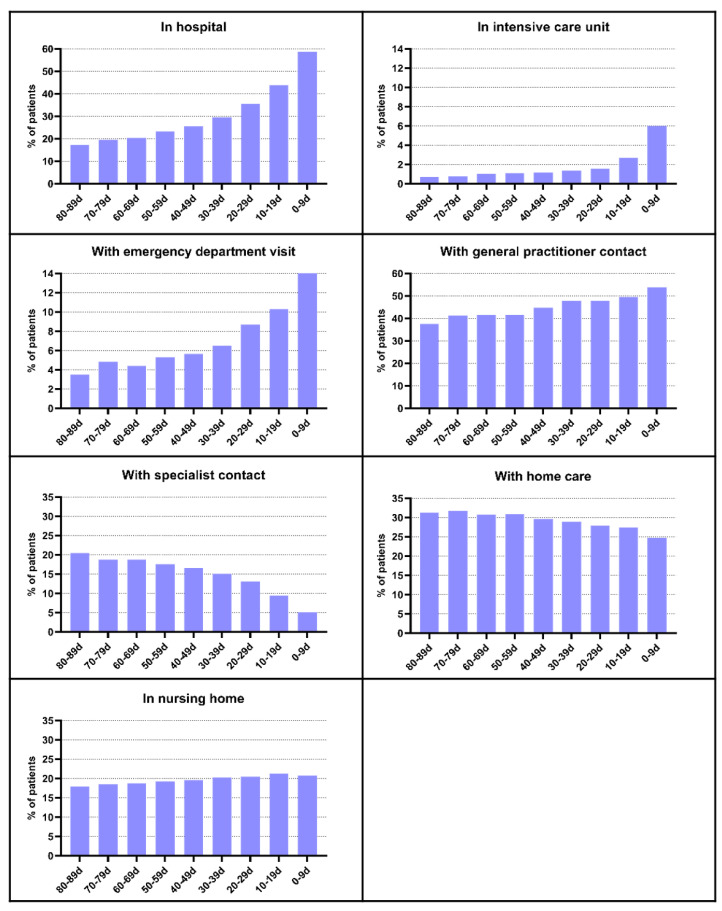
Healthcare utilization at the end of life in older patients with cancer: percentage of patients with use/contacts per 10-day interval in the last three months (*n* = 3546).

The percentage of patients in the hospital, in the ICU, with ED visits, with GP contacts, with specialist contacts, with home care days, and with nursing home days in the last three months of life (per 10-day interval) are displayed in Figure 2. For all these outcomes, the percentage of patients with use or contacts increased throughout the last three months except for specialist contacts and home care days that decreased. The use of inpatient hospital days, ICU days, and ED visits highly increased near death: from 17.3% to 58.6% for hospital days, from 0.7% to 6.0% for ICU days and from 3.5% to 14.6% for ED visits in the last three months. In the last ten days before death (Supplementary Figure S1), the number of patients with hospital admissions, ICU admissions, and GP contacts increased up till death. The other outcomes remained relatively stable in the last ten days. The increase in the last ten days was the highest for hospital admissions, with a rise of 13.8 percentage points. 

Regarding medication use in the last three months before death (Table 3), 29.2% of patients received systemic anti-neoplastic treatment, and 11.9% received endocrine treatment. 56.0% of patients received systemic corticosteroids, 77.7% received opioids, and 30.1% received palliative sedation and/or symptom control with midazolam in the last three months of life. For 23.2% of patients, midazolam was prescribed in the last ten days before death.

### 3.4. Specialized Palliative Care in EOL and Associated Factors

Specialized palliative care (SPC) was received by 1544 patients (43.5%) in the last three months of life (Table 4). SPC consisted of a stay at the hospital palliative care unit (17.0%), palliative home care by a nurse (27.6%) and/or multidisciplinary palliative support team at home (17.2%). A palliative care unit admission lasted a median of 11 days and started a median of 11 days before death. Palliative home care was delivered for a median of 28 days and started a median of 42 days before death. The visits of the multidisciplinary palliative support team started at a median of 22 days before death. 

**Figure 3:**
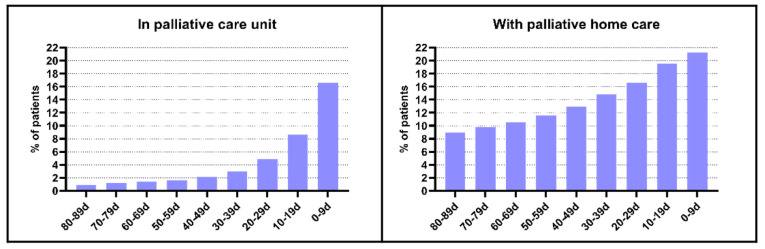
Hospital palliative care and palliative home care use at the end of life in older patients with cancer: percentage of patients with use/contacts (*n* = 3546).

In Figure 3, the percentage of patients with palliative care unit stays and palliative home care increased throughout the last three months of life. In the last ten days, the number of patients admitted to the palliative care unit increased by 6.8 percentage points, while the number of patients with palliative home care stayed relatively stable (Supplementary Figure S2). 

**Figure 4:**
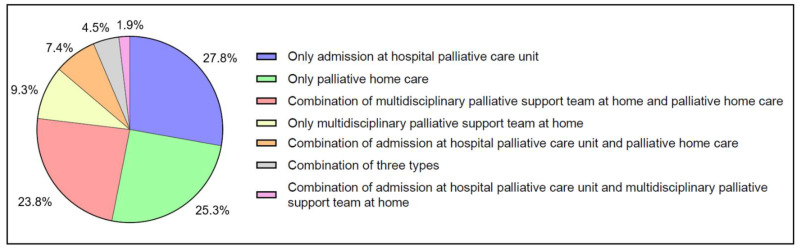
Specialized palliative care use in the last three months before death in older patients with cancer (*n* = 3546). (Mobile palliative care teams in hospitals or nursing homes cannot be distinguished in administrative data and are not reported, potentially leading to an underestimation of the percentage of patients receiving palliative care).

Figure 4 gives details of which type of SPC occured most often. Most patients only had an admission at the hospital palliative care unit (27.8%), only received palliative home care (25.3%), or received a combination of the multidisciplinary palliative support team at home and palliative home care (23.8%). 

Looking at baseline socio-demographic and clinical factors associated with the use of SPC, the odds of using SPC were significantly lower with increasing age, in patients with more comorbidities (CCI), in patients with a poor performance score (ECOG-PS) and in patients that had no partner (Table 5). The odds of using SPC were significantly higher for certain tumor types (colon, lung, and pancreas vs. breast cancer) and with increasing cancer stage. 

The association between GS/GA results at cancer diagnosis and SPC use in the last three months are displayed in Table 6. The G8 score was not associated with using SPC in the last three months. Within the cohort of 2761 patients with an abnormal G8, 43.3% received SPC in the last three months of life. The odds of using SPC were significantly lower in patients with functional dependence (ADL, IADL), a history of falls, and cognitive impairment (MMSE) at cancer diagnosis. The odds of using SPC were significantly higher in patients with pain (VAS) and risk for malnutrition (MNA-SF) at cancer diagnosis. After adjustment for clinical, oncological, and socio-demographic variables, functional dependence based on ADL, IADL and cognitive impairment based on MMSE remained associated with less use of SPC.

### 3.5. Circumstances of Death

Regarding the place of death, 55.0% of patients died in the hospital (16.4% in a palliative care unit, 7.9% in the geriatric department and 30.7% in another department). In total, 17.5% of patients died in a nursing home and 27.6% at home (Table 7). 

The cause of death, as reported in the death certificate, was available for 3102 patients (missing data for deaths in 2018–2019). In the cohort with available data, cancer was the underlying cause of death in 79.3% of patients, and 20.7% died of other causes. Heart failure, chronic obstructive pulmonary disease, and chronic ischemic heart disease were among the most common other causes.

## 4. Discussion

In this cohort of more than 3000 deceased older patients with cancer, the EOL care is intense in the last three months of life with frequent hospital admissions, ED visits and, for some patients, an ICU admission. Furthermore, patients have frequent contact with their GP, use home care services, and one in four has a nursing home stay. This healthcare utilization strongly increases as death approaches, except for home care and nursing home use. Less than half of the patients receive SPC in our study. In patients with potential frailty, functional and cognitive impairment at cancer diagnosis are associated with less SPC use. Eventually, more than half of the older patients with cancer die in the hospital.

Various studies have explored the patterns of care at the EOL in older patients with cancer [21,35,36,37,38,39,40,41,42,43]. All these studies report high rates of healthcare utilization that increase at late stages of life, and some even report aggressive and excessive EOL care [35,40,43]. In studies comparing older patients with cancer to the general older population, high rates of hospitalizations, medicine use and clinician visits in EOL are especially prevalent in the former [44]. Within the older population with cancer, research shows that EOL healthcare utilization decreases with age [35]. Likewise, healthcare utilization (e.g., hospital admissions, ICU admissions, ED visits, and GP contacts) decreased with age in our cohort of 70+ patients.

Directly comparing healthcare utilization across different (international) studies is difficult because of differences in healthcare settings, cohort selection (age cut-off, survival time, cancer diagnosis vs. cancer death, advanced cancer vs. all cancer stages), and follow-up time. Most studies focus on the last month, six months, or 12 months of life [4,21]. One study in the United Kingdom (UK) selecting a three-month observation period before death observed a similar percentage of patients being hospitalized (both 76%), a higher percentage of patients with ED visits (60% in the UK study vs. 50% in our study) and a lower percentage of patients in ICU (4% vs. 11%) [42]. Another study with the same observation period, conducted in the United States (US), reported a higher healthcare utilization for hospitalizations (80% in the US study vs. 76% in our study), ICU (27% vs. 11%), and nursing home stays (31% vs. 24%) [41]. Healthcare utilization thus seems to vary based on country and healthcare setting. A Belgian study by De Schreye et al. reported healthcare and medication use in the last three months before death in patients dying from cancer (all ages, mean age at death 78) [5]. Our study differs in selecting specifically older patients with cancer that died from any cause and in having clinical and population-based data available. Compared to our research, the percentage of patients hospitalized (79% in the study of De Schreye et al. vs. 76% in our study), patients with ED visits (53% vs. 49%), and patients with opioids use (76% vs. 78%) were relatively similar. Only the percentage of patients with systemic anti-neoplastic therapy was lower in our cohort (36% vs. 29%). The selection of older patients dying from all causes in our cohort might be the reason for this lower use. Overall, these findings on EOL healthcare utilization suggest a substantial dependence on hospitals to provide EOL care (increasing hospitalizations, ICU admissions, and ED visits vs. decreasing home care use).

Regarding using SPC in the last three months of life, this was observed in 44% of our cohort of deceased older patients with cancer. Although this percentage is difficult to compare internationally because of differences in healthcare organizations for SPC, a review on EOL healthcare utilization in cancer care found percentages ranging between 51–57% for older patients in the US [4]. A study in the UK (all ages, 80% aged 70+) reports that 64.6% of patients with advanced cancer received palliative care [45]. Compared with Belgian data from De Schreye et al., 47% of patients dying from cancer received SPC [5]. The lower percentage in our cohort could be explained by the selection of older patients that died of all causes. Various studies have reported that older patients with cancer receive less palliative care [4,23,46]. Like De Schreye et al., we were, however, unable to capture mobile palliative care teams in hospitals or nursing homes using healthcare reimbursement data. Another Belgian study (including all ages, 86% age 65+) on providing palliative care services reported 61% of palliative care use in cancer deaths based on a national representative network of GPs [47]. In the latter, mobile palliative care teams in hospitals and nursing homes were included and confirmed the underestimation in our study that used healthcare reimbursement data (that doesn’t contain information on mobile palliative care teams or in nursing homes). Nevertheless, comparing these results, our study indicates that SPC in a community setting, and a palliative hospital unit might be underutilized by older patients with cancer. 

SPC was started a median of 30 days before death in our cohort. An international review on the duration of palliative care reports that palliative care started a median of 15 days before death in patients with cancer [48]. In a Belgian study of the general older population dying of non-sudden death, palliative care had also started a median of 15 days before death [49]. Our older population with cancer thus received SPC earlier than internationally reported and the general older population in Belgium. A timely start is important for the full benefits of SPC to be realized [48]. However, evidence suggests that SPC should be started even earlier, i.e., 3–4 months before death and should be integrated alongside standard oncologic care for incurable cancer [7,48]. In our cohort, SPC is still mostly considered in the terminal phase, confirming that also in Belgian clinical practice, early integration of SPC has yet to be achieved. In Belgian law, reimbursement for palliative patients is also linked to a prognosis of three months. Research has, however, shown that more than half of Belgian palliative patients received home-based specialized palliative care more than three months before death. Nevertheless, we believe the law should not only focus on this terminal phase but better align with the evidence on the timely start of palliative care [12].

Which factors are associated with SPC use in the EOL has been studied in older patients with cancer [23,46]. In line with our results, SPC use was lower for patients who were older, who lived alone and who had comorbidities. SPC use was higher in patients with more advanced cancer stages and patients with lung and colon cancer [23,46]. Various studies have also shown that individuals with a lower education level are less likely to receive palliative care, which is not supported by our results [46]. These studies are mostly from the US. Compared to the US, the Belgian healthcare system is more equally accessible. Belgium has compulsory health insurance at low cost, which could explain the lack of association between attained educational level and SPC use (that was also not found in the Belgian study by De Schreye et al. [5]). 

Disparities in palliative care use have thus been shown, but why specifically, older patients use less palliative care is not fully understood. A possible reason quoted is that palliative care may be delivered by geriatricians and other internal medicine physicians in the hospital setting for older patients, which is often not captured [23]. This may also have been the case in our study since ‘basic’ palliative care was not captured in this study [50]. Another reason that may account for less specialized palliative care is that physicians perceive older patients as experiencing less symptom distress and maybe in less need of expert symptom control and psychological support offered by SPC services [51]. Frailty and comorbidity might play a role by causing hurdles in clinical assessments, treatment decisions and EOL care planning. Frailty is a multidimensional syndrome with possible deficits in mobility, nutrition, cognition, functional status, mood etc. To our knowledge, which dimensions of frailty are associated with less SPC use had never been studied in older patients with cancer. The use of GA to assess frailty in the palliative setting for older patients with cancer is generally poorly investigated [52]. With our data, we were able to demonstrate that patients with functional dependence (ADL, IADL) and cognitive impairment (MMSE) at cancer diagnosis are less likely to receive SPC even after adjustment for confounders. Specifically, the cognitive and functional status might thus play a role in referral to SPC. Little research is available on this topic. Two studies have shown that patients with cancer and dementia had longer hospital stays, more intensive care unit stays and receive less palliative care in EOL [53,54]. One US study found a contrasting result for functional status, i.e., that patients with functional impairment had higher odds of receiving an inpatient palliative care consultation and being discharged to hospice in hospitalized patients with advanced cancer [55]. An abnormal G8 score at cancer diagnosis indicating potential frailty was not associated with SPC use. However, in the older population, frailty has been associated with less access to palliative care [56]. These discrepancies might be because of differences in healthcare systems and differences between the general older population and the older population with cancer. The G8 tool is not a diagnostic tool for frailty but merely a geriatric screening tool to identify patients needing a GA. Our results did, however, indicate that certain characteristics of frailty (i.e., functional, and cognitive status) might pose barriers to providing SPC in the community setting or palliative care unit. The relationship between frailty and SPC use in older cancer patients is complex, and more prospective research is needed. 

Patients who reside in a nursing home at baseline often have functional and cognitive impairment at cancer diagnosis (this is also the case in our cohort). These patients will have less chance of (multidisciplinary) palliative home care and palliative care unit admission (SPC) in the EOL period because of their living situation. Therefore, we also performed a sensitivity analysis by adding baseline nursing home resident status as a covariate in the multi-variable model (Supplementary Table S6). Baseline functional (IADL) and cognitive (MMSE) impairment remained significantly associated with less SPC use in the EOL regardless of if patients were nursing home residents or not at baseline. 

Place of death is an established indicator of the quality of EOL care and has been investigated in a multitude of studies in older patients with cancer [57]. In our study, 55% of patients died in the hospital (39% of patients died in a non-palliative care unit). Given the fact that older patients with cancer even more so prefer dying at home, this is a high number [58]. Hospital death has also been associated with higher healthcare service use and cost in the EOL period [59,60]. In a large US study of older patients dying of cancer, 36% died in the hospital or hospice and 19% in a nursing home [57]. In the Belgian study by De Schreye et al., 61% of patients died in the hospital [5]. Older patients with cancer seem to die more often outside of the hospital compared to the general population with cancer, but the percentage of hospital deaths remains high. 

In our cohort, cancer was reported as the cause of death in 79% of patients. A Norwegian study of older patients with a history of cancer found that 81% of patients died because of cancer [23]. However, the accuracy of cause-of-death data derived from death certificates has been disputed, and in older adults, accuracy is estimated to be even lower [61]. Therefore, we included patients with a new cancer diagnosis who died from any cause. Furthermore, a history of cancer might play a role in the healthcare utilization at EOL even though the patient didn’t die from cancer. 

A major strength of our study is the large sample size with a representative picture of older patients with various cancers, as seen in Belgian oncology practice. Furthermore, a unique linkage was achieved between clinical data from a prospective study and population-based data, allowing the exploration of associations between the two and a long-term follow-up. Starting from a previous clinical study also gives another perspective compared to studies selecting patients that die from cancer. Clinicians cannot predict who will die from cancer, making the results more applicable to clinical practice. Nevertheless, our study has some limitations. By using healthcare reimbursement data for studying outcomes, we have no information on whether certain healthcare or medication use was appropriate or not and what the patient’s preference was. Healthcare reimbursement data are not collected for research purposes, administrative errors may occur, and there may be a financial incentive which might impact the validity. Another consequence is that we could not identify mobile palliative care teams in the hospital, mobile palliative care teams in the nursing home, and basic palliative care within non-palliative care units like geriatric care units, leading to an underestimation of palliative care use.

Furthermore, we included a heterogeneous sample of patients regarding survival time, ranging from patients diagnosed with cancer three months before they died to patients diagnosed up to nine years before death. This means that GS/GA data might not reflect the general health status of patients in the last three months of life and that the impact of the cancer diagnosis might vary for patients. Therefore, we adjusted for the time between GS/GA and death. Ideally, we would have GS/GA data (and other clinical and sociodemographic data) from 3 months before death, but understandably, this can only be determined retrospectively. Data from the time of cancer diagnosis is a more feasible alternative and represents a clearly defined time point in the disease trajectory. In addition, other potential confounding factors (such as the presence of an informal caregiver, household income or whether the patient still received oncological treatment in EOL) are not captured. Possible trends in healthcare use (including SPC use) during the study period were not explored in this manuscript. Finally, our findings are based on a particular population of those who died at least three months after cancer diagnosis and, therefore, cannot be generalized to those who die soon after diagnosis. 

Future studies should focus on optimising healthcare utilization in the last phase of life in older patients with cancer. The association between cognitive or functional impairment and SPC use must be further explored in retrospective and prospective studies. If causal evidence is found, ways to broaden access to SPC for these patients should be studied. The integration of GS/GA into palliative care for older patients with cancer also needs to be explored further to evaluate the possible benefits of GS/GA in the EOL phase.

## 5. Conclusions

Using linked datasets, this study gives insight into the final phase of life of older patients with cancer and demonstrates which factors may influence access to SPC in this population. We found a high healthcare utilization with a substantial dependence on hospital care (hospital admissions, ICU admissions and ED visits) in the last three months of life. There was an increasing trend of healthcare utilization near death, and more than half of the patients died in the hospital. Furthermore, we demonstrated that patients with functional and cognitive impairment at cancer diagnosis are less likely to receive SPC in Belgium.

## Figures and Tables

**Table 1:** Patient Characteristics.

		Full Cohort (Normal and Abnormal G8 Score)	Cohort with GA Data (Only Abnormal G8 Score)
		N (%)	N (%)
Total		3546 (100.0)	2761 (100.0)
Age	70–74 y	883 (24.9)	598 (21.7)
	75–79 y	1039 (29.3)	737 (26.7)
	80–84 y	939 (26.5)	783 (28.4)
	≥85 y	685 (19.3)	643 (23.3)
	Median (IQR)	79 (75–83)	80 (75–84)
Sex	Female	1902 (53.6)	1494 (54.1)
	Male	1644 (46.4)	1267 (45.9)
Cancer site	Breast	655 (18.5)	466 (16.9)
	Colon	536 (15.1)	440 (15.9)
	Lung	514 (14.5)	394 (14.3)
	Rectum	215 (6.0)	167 (6.0)
	Pancreas	152 (4.3)	134 (4.9)
	Other ^a^	1474 (41.6)	1160 (42.0)
Cancer stage ^b^	I	399 (11.2)	290 (10.5)
	II	702 (19.8)	544 (19.7)
	III	742 (20.9)	563 (20.4)
	IV	956 (27.0)	765 (27.7)
	NA ^c^	456 (12.9)	361 (13.1)
	Unknown	291 (8.2)	238 (8.6)
G8	Normal	752 (21.2)	0 (0.0)
	Abnormal	2794 (78.8)	2761 (100.0)
CCI	No comorbidity: score 0	1015 (28.6)	728 (26.4)
	Mild comorbidity: score 1–2	1446 (40.8)	1143 (41.4)
	Moderate comorbidity: score 3–4	722 (20.4)	588 (21.3)
	Severe comorbidity: score ≥ 5	341 (9.6)	285 (10.3)
	Missing	22 (0.6)	17 (0.6)
Polypharmacy	0–4 medications	1520 (42.9)	1042 (37.7)
	≥5 medications	1962 (55.3)	1675 (60.7)
	Missing	64 (1.8)	44 (1.6)
ECOG-PS	Score 0–1	2229 (62.9)	1514 (54.8)
	Score ≥ 2	1307 (36.8)	1246 (45.1)
	Missing	10 (0.3)	1 (0.0)
Attained educational level	Higher education	587 (16.5)	456 (16.5)
	Upper secondary education	958 (27.0)	749 (27.1)
	Lower secondary education	1388 (39.1)	1108 (40.1)
	Primary education	393 (11.1)	346 (12.5)
	Illiterate	27 (0.8)	26 (1.0)
	Other	34 (1.0)	30 (1.1)
	Missing	159 (4.5)	46 (1.7)
Marital status	Partnered (married or legally co-habiting)	1810 (51.0)	1392 (50.4)
	Not partnered (single, widow/er, divorced)	1611 (45.4)	1351 (48.9)
	Other	20 (0.6)	16 (0.6)
	Missing	105 (3.0)	2 (0.1)
Cohort identification ^d^	Study 1	593 (16.7)	471 (17.1)
	Study 2	444 (12.5)	328 (11.9)
	Study 3	2509 (70.8)	1962 (71.1)
Time between GS/GA and death in years	3 months–1 year ^e^	1211 (34.1)	1030 (37.3)
	1–2 years	878 (24.8)	698 (25.3)
	2–3 years	533 (15.0)	396 (14.3)
	3–4 years	364 (10.3)	249 (9.0)
	4–5 years	284 (8.0)	195 (7.1)
	>5 years	276 (7.8)	193 (7.0)
	Median (IQR)	1.6 (0.7–3.1)	1.4 (0.7–2.9)

^a^ Other: frequencies ≤150 in the full cohort are categorized under other (a more exhaustive list of tumor types is available in Supplementary Table S5). ^b^ Combined stage (created for this study): the pathological stage prevails over the clinical stage, except for cases with clinical stage IV, cases with missing pathological stage or pathological stage defined after neo-adjuvant treatment. ^c^ NA: TNM staging is not applicable for certain tumor sites (e.g., tumors of the central nervous system) or morphology codes (e.g., angiosarcoma). ^d^ Patients were selected from three consecutive multicentric prospective observational cohort studies (October 2009 to February 2015). Cohort identification specifies which study. ^e^ Patients were included if they survived at least three months after the performance of GS/GA to allow a full 3-month observation period (cf. methods). (Abbreviations: GA, geriatric assessment; IQR, interquartile range; CCI, Charlson Comorbidity Index; NA, not applicable; ECOG-PS, Eastern Cooperative Oncology Group Performance Status; GS, geriatric screening).

**Table 2:** Healthcare utilization in the last three months before death in older patients with cancer (*n* = 3546).

	N (%)	Median Number in Last 3 m (In Case of Use) (IQR)
Hospital admission	2705 (76.3)	20 days (9–36)
ICU admission	371 (10.5)	4 days (2–8)
ED visits	1743 (49.1)	1 visit (1–2)
GP contacts ^a^	3148 (89.9)	6 contacts (3–10)
Specialist contacts	2147 (60.5)	2 contacts (1–3)
Home care use	1895 (53.4)	26 days (8–58)
Nursing home stay	856 (24.1)	83 days (53–90)

^a^ Patients (*n* = 46) registered in community health centers are excluded as the number of GP contacts cannot be distinguished. (Abbreviations: ICU: intensive care unit, ED: emergency department, GP: general practitioner.)

**Table 3:** Medication use in the last three months before death in older patients with cancer (*n* = 3546).

Medication Use	N (%)
Systemic anti-neoplastic therapy	1038 (29.2)
Endocrine therapy	422 (11.9)
Systemic corticosteroids	1985 (56.0)
Opioids	2757 (77.7)
Midazolam	1066 (30.1)

**Table 4:** Specialized palliative care use in the last three months before death in older patients with cancer (*n* = 3546).

	N (%)	Median Number of Days in Last 3 m (In Case of Use) (IQR)	Median Start in Days before Death (IQR)
Specialized palliative care ^a^	1544 (43.5)	-	30 (11–74)
Palliative care unit admission	604 (17.0)	11 (5–24)	11 (5–25)
Palliative home care	979 (27.6)	28 (11–64)	42 (15–88)
Multidisciplinary palliative support team at home	610 (17.2)	-	22 (8–43)
No specialized palliative care	1992 (56.5)	-	-

^a^ Mobile palliative care teams in hospitals or nursing homes cannot be distinguished in administrative data and are not reported, potentially leading to underestimation of the percentage of patients receiving palliative care.

**Table 5:** Logistic regression presenting the association between baseline patient/clinical/socio-demographic factors at new cancer diagnosis and subsequent use of specialized palliative care in the last three months of life in older patients with cancer (*n* = 3546).

		OR (95% CI)	*p*-Value	Type III *p*-Value
Age category	70–74 y	1		**<0.001**
	75–79 y	0.90 (0.75–1.08)	0.25	
	80–84 y	0.71 (0.59–0.85)	**<0.001**	
	≥85 y	0.44 (0.36–0.54)	**<0.001**	
Sex	Female	1		0.08
	Male	1.13 (0.99–1.29)	0.08	
Cancer site	Breast	1		**<0.001**
	Colon	1.37 (1.08–1.74)	**0.009**	
	Lung	2.36 (1.86–2.99)	**<0.001**	
	Rectum	1.10 (0.80–1.53)	0.55	
	Pancreas	4.12 (2.83–5.99)	**<0.001**	
	Other ^a^	1.31 (0.96–1.79)	0.09	
Cancer stage ^b^	I	1		**<0.001**
	II	1.21 (0.93–1.58)	0.16	
	III	1.92 (1.48–2.49)	**<0.001**	
	IV	3.46 (2.69–4.45)	**<0.001**	
	NA ^c^	1.74 (1.31–2.31)	**<0.001**	
	Missing	2.22 (1.62–3.05)	**<0.001**	
Charlson comorbidity index	No comorbidity: score 0	1		**0.0013**
	Mild comorbidity: score 1–2	0.91 (0.78–1.07)	0.25	
	Moderate comorbidity: score 3–4	0.78 (0.64–0.95)	**0.01**	
	Severe comorbidity: score ≥ 5	0.62 (0.48–0.78)	**<0.001**	
	Missing	1.36 (0.58–3.19)	0.47	
Polypharmacy	0–4 medications	1		**<0.001**
	≥5 medications	0.72 (0.63–0.82)	**<0.001**	
	Missing	0.79 (0.47–1.30)	0.35	
ECOG-PS	Score 0–1	1		**<0.001**
	Score ≥ 2	0.69 (0.60–0.79)	**<0.001**	
	Missing	0.75 (0.21–2.68)	0.66	
Attained educational level	Higher education	1		0.40
	Upper secondary education	0.90 (0.73–1.10)	0.31	
	Lower secondary education	0.98 (0.81–1.19)	0.82	
	Primary education	0.88 (0.68–1.14)	0.33	
	Illiterate	1.31 (0.61–2.83)	0.49	
	Other	1.1 (0.54–2.16)	0.83	
	Missing	0.70 (0.49–1.00)	0.05	
Marital status	Partnered	1		**<0.001**
	Not partnered	0.61 (0.53–0.70)	**<0.001**	
	Other	0.18 (0.05–0.61)	**0.006**	
	Missing	0.60 (0.40–0.90)	**0.01**	
Cohort identification ^d^	Study 1	1		**0.1521**
	Study 2	1.17	0.23	
	Study 3	1.20	0.05	
The time between G8 and death in days ^e^	90–267 ^f^	1		**<0.001**
	268–568	1.21 (1.01–1.46)	**0.04**	
	569–1135	0.93 (0.77–1.12)	0.44	
	>1135	0.61 (0.51–0.74)	**<0.001**	

^a^ Other: frequencies ≤150 in the full cohort are categorized under other (a more exhaustive list of tumor types is available in Supplemental Table S5). ^b^ Combined stage (created for this study): the pathological stage prevails over the clinical stage, except for cases with clinical stage IV, cases with missing pathological stage or pathological stage defined after neo-adjuvant treatment. ^c^ NA: TNM staging is not applicable for certain tumor sites (e.g., tumors of the central nervous system) or morphology codes (e.g., angiosarcoma). ^d^ Patients were selected from three consecutive multicentric prospective observational cohort studies (October 2009 to February 2015). Cohort identification specifies which study. ^e^ 4 categories based on Q1, median and Q3. ^f^ Patients were included if they survived at least three months after performing G8 (and GA) to allow a full 3-month observation period (cf. methods). Bold numbers indicate *p*-values < 0.05. (Abbreviations: ECOG-PS: Eastern Cooperative Oncology Group Performance Status; NA: not applicable; G8: Geriatric 8).

**Table 6:** Logistic regression presenting the association between baseline GS/GA results at new cancer diagnosis and subsequent use of specialized palliative care in the last three months of life in older patients with cancer.

		Unadjusted Analysis		Adjusted Analysis ^a^	
		OR (95% CI)	*p* Value	OR (95% CI)	*p* Value
Geriatric screening at cancer diagnosis (*n* = 3546)					
G8	Normal vs. abnormal	0.93 (0.79–1.09)	0.3803	0.94 (0.78–1.14)	0.5412
Geriatric assessment at cancer diagnosis (*n* = 2761)					
Functional status: ADL	Dependent vs. independent	0.66 (0.57–0.77)	**<0.0001**	0.82 (0.69–0.96)	**0.0172**
Functional status: IADL ^b^	Dependent vs. independent	0.64 (0.55–0.75)	**<0.0001**	0.76 (0.64–0.91)	**0.0028**
Falls history ^b^	Falls vs. no falls	0.77 (0.66–0.90)	**0.0011**	0.88 (0.74–1.04)	0.1319
Pain: VAS ^b^	Pain vs. no pain	1.21 (1.04–1.40)	**0.0156**	1.15 (0.98–1.36)	0.0933
Cognition: MMSE ^b^	Mild/severe cognitive impairment vs. normal	0.64 (0.53–0.78)	**<0.0001**	0.73 (0.59–0.91)	**0.0046**
Depression: GDS-4 ^b^	At risk for depression vs. not at risk	0.96 (0.82–1.12)	0.6127	0.89 (0.76–1.06)	0.1963
Nutrition: MNA-SF ^b^	At risk for malnutrition/malnourished vs. normal	1.39 (1.14–1.70)	**0.0013**	1.04 (0.84–1.30)	0.7062

^a^ Adjusted for age, sex, tumor type, stage, Charlson Comorbidity Index, attained educational level, marital status, cohort identification, and time between GA and death. ^b^ Patients with missing data were omitted (the number of patients with missing data is displayed in Supplementary Table S1). Bold numbers indicate *p*-values < 0.05. (Abbreviations: GS: geriatric screening; GA: geriatric assessment; OR: odds ratio; CI: confidence interval; G8: Geriatric 8; ADL: Activities of Daily Living; IADL: Instrumental Activities of Daily Living; VAS: Visual Analogue Scale; MMSE: Mini-Mental State Examination; GDS-4: 4-item Geriatric Depression Scale; MNA-SF: Mini Nutritional Assessment-Short Form).

**Table 7:** Place of death in older patients with cancer (*n* = 3546).

Place of Death	N (%)
Hospital: palliative care unit	583 (16.4)
Hospital: geriatric department	279 (7.9)
Hospital: other department	1087 (30.7)
Nursing home	620 (17.5)
Home	977 (27.5)

## Data Availability

The linked data of the current study is not publicly available because of data protection and security reasons.

## Supplementary Materials

The following supporting information can be downloaded at: https://www.mdpi.com/article/10.3390/cancers15133349/s1, Table S1: Geriatric assessment content, cut off scores and results (*n* = 2761); Table S2: Definition of outcomes identified in healthcare reimbursement data from the Belgian InterMutualistic Agency (IMA); Table S3: Outcomes and corresponding nomenclature codes used in healthcare reimbursement data from the Belgian InterMutualistic Agency (IMA); Table S4: Medication and corresponding ATC code used in healthcare reimbursement data from the Belgian InterMutualistic Agency (IMA); Table S5: Detailed list of tumor types; Figure S1: Healthcare utilization at the end of life in older patients with cancer: percentage of patients with use/contacts in the last 10 days of life (*n* = 3546); Figure S2: Hospital palliative care and palliative home care use at the end of life in older patients with cancer: percentage of patients with use/contacts in the last 10 days (*n* = 3546); Table S6: Sensitivity analysis (baseline nursing home resident status added as co-variate): Logistic regression presenting the association between baseline GS/GA results at new cancer diagnosis and subsequent use of specialized palliative care in the last 3 months of life in older patients with cancer. (References [62,63,64,65,66,67,68] are cited in the Supplementary Materials).
