# Supplementary material for: End-of-Life Care in the Last Three Months before Death in Older Patients with Cancer in Belgium: A Large Retrospective Cohort Study Using Data Linkage
Source: Cancers (Basel). 2023 Jun 26;15(13):3349. doi: 10.3390/cancers15133349 (PMC10340177; doi:10.3390/cancers15133349)
Supplement: Supplementary file 1 [file cancers-15-03349-s001.zip › cancers-2440156-supplementary.pdf]

Supplementary Materials

Table of Contents:

Table S1: Geriatric assessment content, cut off scores and results (n=2,761) 2

Table S2: Definition of outcomes identified in healthcare reimbursement data from the Belgian InterMutualistic Agency (IMA) 3

Table S3: Outcomes and corresponding nomenclature codes used in healthcare reimbursement data from the Belgian InterMutualistic Agency (IMA) 4

Table S4: Medication and corresponding ATC code used in healthcare reimbursement data from the Belgian InterMutualistic Agency (IMA) 5

Table S5: Detailed list of tumor types 6

Figure S1: Healthcare utilization at the end of life in older patients with cancer: percentage of patients with use/contacts in the last 10 days of life (n=3,546) 7

Figure S2: Hospital palliative care and palliative home care use at the end of life in older patients with cancer: percentage of patients with use/contacts in the last 10 days (n=3,546) 8

Table S6: Sensitivity analysis (baseline nursing home resident status added as co-variate): Logistic regression presenting the association between baseline GS/GA results at new cancer diagnosis and subsequent use of specialized palliative care in the last 3 months of life in older patients with cancer . 9

Table S1: Geriatric assessment content, cut off scores and results (n=2,761)

Domain	Scale	Operationalization	N (%)
Functional status	Katz's Activities of Daily Living (ADL) [6-24] [1]	Independent: score = 6	1158 (41.9)
		Dependent: score ≥ 7	1603 (58.1)
		Missing	0
	Lawton's Instrumental Activities of Daily Living (IADL)[a] [0-5/0-8] [2]	Independent: score 5 (male) or 8 (female)	905 (33.0)
		Dependent: score < 5 (male) or < 8 (female)	1834 (67.0)
		Missing	22
Falls history	Number of falls in the past 12 months [3]	No falls: 0	1709 (62.3)
		Presence of falls: ≥ 1	1035 (37.7)
		Missing	17
Pain	Visual Analogue Scale [0-10] [4]	No pain: score = 0	1370 (50.6)
		Presence of pain: score ≥ 1	1035 (49.4)
		Missing	54
Cognition	Mini Mental State Examination (MMSE) [0-30] [5]	Normal cognition: score ≥ 24	2010 (78.3)
		Mild/severe cognitive impairment: score < 24	556 (21.7)
		Missing	195
Depression	4-item Geriatric Depression Scale (GDS-4) [0-4] [6]	Not at risk for depression: score 0	1118 (42.9)
		At risk for depression: score ≥ 1	1488 (57.1)
		Missing	155
Nutrition	Mini Nutritional Assessment – Short Form (MNA-SF) [0-14] [7]	Normal nutrition: score ≥ 12	490 (17.8)
		Risk for malnutrition/malnourished: score < 12	2262 (82.2)
		Missing	9

[a] The Lawton IADL scale includes eight items but some of these items (cooking, housekeeping, and doing laundry) are mostly only applicable in women. Therefore, these three items were not assessed in males in the original form.

References:

1. Katz S, Ford AB, Moskowitz RW, Jackson BA, Jaffe MW. Studies of Illness in the Aged. The Index of Adl: A Standardized Measure of Biological and Psychosocial Function. JAMA. 1963;185:914-9.
2. Lawton MP, Brody EM. Assessment of older people: self-maintaining and instrumental activities of daily living. Gerontologist. 1969;9:179-86.
3. Lamb SE, Jørstad-Stein EC, Hauer K, Becker C. Development of a common outcome data set for fall injury prevention trials: the Prevention of Falls Network Europe consensus. J Am Geriatr Soc. 2005;53:1618-22.
4. Collins SL, Moore RA, McQuay HJ. The visual analogue pain intensity scale: what is moderate pain in millimetres? Pain. 1997;72:95-7.
5. Folstein MF, Folstein SE, McHugh PR. "Mini-mental state". A practical method for grading the cognitive state of patients for the clinician. J Psychiatr Res. 1975;12:189-98.
6. Yesavage JA, Brink TL, Rose TL, Lum O, Huang V, Adey M, et al. Development and validation of a geriatric depression screening scale: a preliminary report. J Psychiatr Res. 1982;17:37-49.
7. Rubenstein LZ, Harker JO, Salvà A, Guigoz Y, Vellas B. Screening for undernutrition in geriatric practice: developing the short-form mini-nutritional assessment (MNA-SF). J Gerontol A Biol Sci Med Sci. 2001;56:M366-72.

Table S2: Definition of outcomes identified in healthcare reimbursement data from the Belgian InterMutualistic Agency (IMA)

Outcome	Description
Hospital admission	Any day spent in an acute hospital for an all-cause inpatient admission
Emergency department (ED) visits	Any day with a contact with an emergency doctor in an emergency department of an acute hospital
Intensive care unit (ICU) admission	Any day spent at the intensive care department of an acute hospital
General practitioner (GP) contacts [a]	Any day with at least one contact (consultation/visit) with a general practitioner
Specialist contacts	Any contact (consultation/visit) with a specialist doctor
Home care use	Any day with technical nursing intervention or hygienic care administered by a professional nurse (excluding specialized palliative care by nurse)
Nursing home (NH) stay	Any day spent at a long-term residential facility with professional nursing care
In nursing home at baseline	NH admission at baseline OR hospital admission at baseline but NH admission ends day of or day before start hospital admission
Palliative care unit admission	Any day spent at the palliative care unit of an acute hospital
Palliative home care use	Any day with nursing care and support for the palliative patient at home
Multidisciplinary palliative support team at home	Any lump sum billed for the provision of advice and coordination of specialized palliative care for primary care providers and provision of psychological support to the patient and informal caregivers

[a] Patients (n=46) registered in a community health center are excluded because lump sum fee covers different primary caregivers in one center so GP cannot be distinguished

Table S3: Outcomes and corresponding nomenclature codes used in healthcare reimbursement data from the Belgian InterMutualistic Agency (IMA)

Outcome	Nomenclature code in Belgian healthcare system
Hospital admission	Not applicable, derived from IMA hospital database
Intensive care unit (ICU) admission	Not applicable, selected based on department code 490 for intensive care unit of hospital
Geriatric department	Not applicable, selected based on department code 300 for geriatric department of hospital
Emergency department (ED) visits	590516 590531 590553 590575 590590 590612 590634 590656 590671 590693 590715 590730 590752 590774 590796 590811
General practitioner (GP) [a] contacts	101010 101032 101076 103110 103132 103213 103235 103412 103434 104215 104230 104252 104510 104532 104554 109723 109045 109060 109082 109701 104370 104392 104414 104436 104451
Specialist contacts	101275 101290 102012 102034 102093 102115 102130 102152 102174 102196 102211 102233 102255 102270 102292 102314 102336 102535 102550 102594 102616 102631 102653 102675 102690 102712 102734 102756 102815 102830 102874 102896 102911 102933 102955 102970 102992 103250 103014 103051 103073
Home care sue	421072 421094 423054 423076 423091 423113 423135 423150 423172 423194 423216 423231 423253 423275 423290 423312 423334 424255 424270 424292 424314 424336 424351 424373 424395 424410 424432 424454 424476 424491 424513 424535 424874 424896 424933 424955 425014 425036 425051 425110 425176 425191 425213 425272 425294 425316 425375 425412 425434 425456 425515 425574 425596 425611 425670 425692 425714 425736 425751 425773 427416 427431 427475 427490 427534 427556 428035 428050 429015
Nursing home (NH) stay	760233 760255 760270 760292 760314 760336 760351 760373 760395 760410 760432 760454 760476 760476 760675 760690 760712 760734 760756 763033 763055 763070 763092 763114 763136 763151 763173 763195 763210 763232 763254 763276 763291 763313 763335 763350 763372 763696 763711
Palliative care unit admission	597763 599782 599804 768143 768445 768762 768784 768806 768821
Palliative home care use	426510 426532 426554 426871 426893 426915 426930 426952 427011 427033 427055 427070 427092 427114 427136 427151 427173 427195
Multidisciplinary palliative support team at home	774056 774071 784092

[a] Patients registered in community health center (nomenclature code: 109616) are excluded because lump sum fee covers different primary caregivers in one center so GP cannot be distinguished.

Table S4: Medication and corresponding ATC code used in healthcare reimbursement data from the Belgian InterMutualistic Agency (IMA)

Medication	ATC code
Systemic anti-neoplastic treatment	L01
Endocrine treatment [a]	L02AB01 L02AE01 L02AE02 L02AE03 L02AE04 L02AE05 L02BA01 L02BA02 L02BA03 L02BB01 L02BB02 L02BB03 L02BB04 L02BG03 L02BG04 L02BG06 L02BX02 G03XC01 G03HA01 G04CB02
Systemic corticosteroids	H02
Opioids	N02A
Palliative sedation with midazolam	N05CD08

[a] For endocrine treatment a specific list of medication validated by the Belgian Cancer Registry is used that contains hormonal agents used for oncologic treatment of breast and prostate cancer in Belgium

Table S5: Detailed list of tumor types

	**Full cohort (n=3,546)**	**Cohort with GA data (n=2,761)**
**Tumor types**	**N (%)**	**N (%)**
Breast	655 (18.5)	466 (16.9)
Colon	536 (15.1)	440 (15.9)
Lung	514 (14.5)	394 (14.3)
Rectum	215 (6.1)	167 (6.1)
Pancreas	152 (4.3)	134 (4.9)
Esophagus	140 (4.0)	120 (4.4)
Non-Hodgkin lymphoma	134 (3.8)	107 (3.9)
Bladder	122 (3.4)	99 (3.6)
Ovary	117 (3.3)	88 (3.2)
Head and neck	108 (3.1)	82 (3.0)
Prostate	104 (2.9)	72 (2.6)
Stomach	76 (2.1)	61 (2.2)
Corpus uteri	75 (2.1)	56 (2.0)
Multiple myeloma	70 (2.0)	55 (2.0)
Liver	53 (1.5)	40 (15)
Kidney	51 (1.4)	39 (1.4)
Unknown primary site	46 (1.3)	44 (1.6)
Gallbladder and bile ducts	43 (1.2)	37 (1.3)
Central nervous system	37 (1.0)	30 (1.1)
Melanoma	31 (0.9)	20 (0.7)
Acute myeloblastic leukemia	21 (0.6)	14 (0.5)
Cervix uteri	20 (0.6)	17 (0.6)
Other [a]	226 (6.4)	179 (6.5)

[a] Other (frequencies <20 in the full cohort)

Figure S1: Healthcare utilization at the end of life in older patients with cancer: percentage of patients with use/contacts in the last 10 days of life (n=3,546)

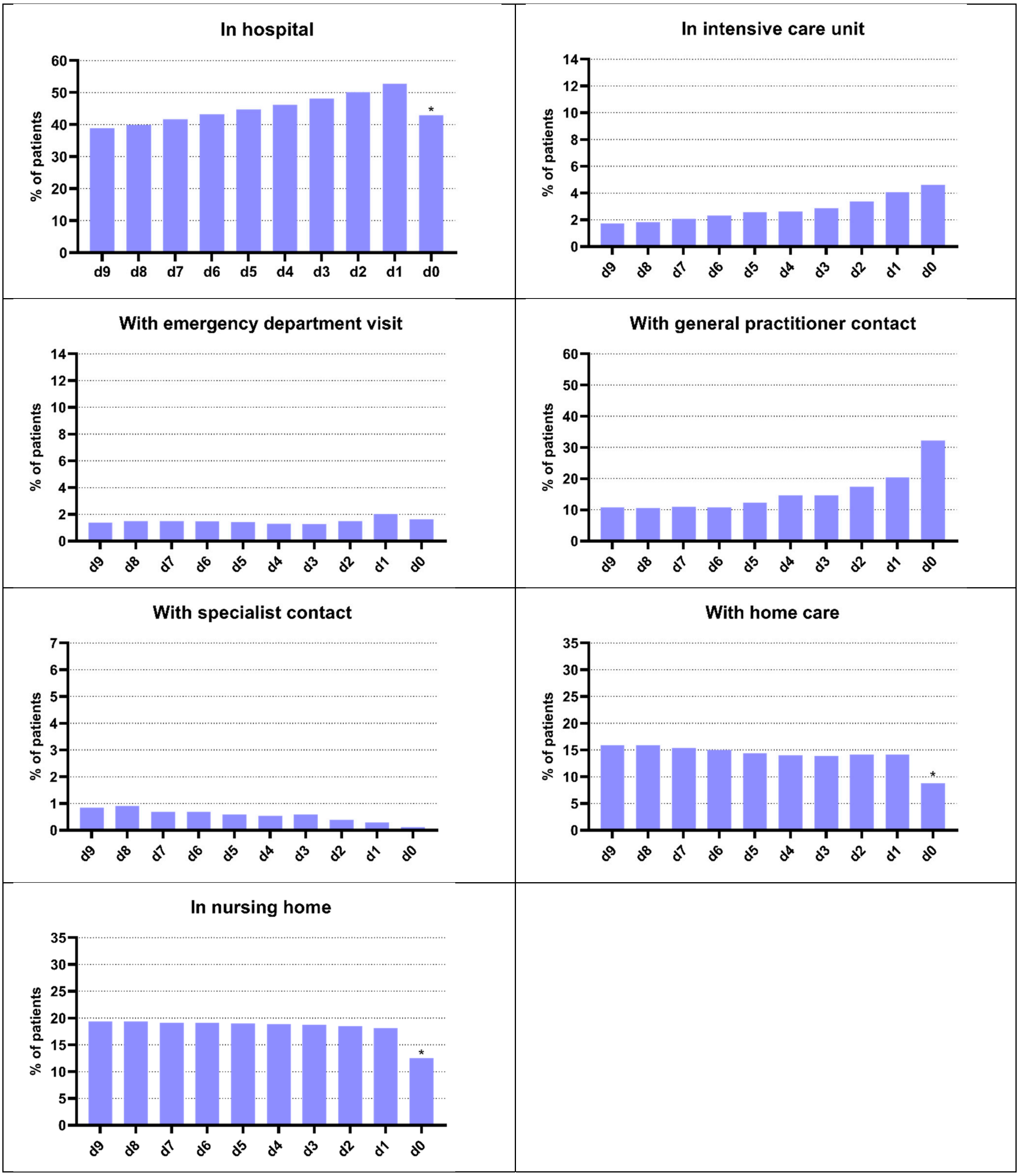

* Because of billing rules, % of patients with use on the day of death might be an underestimation because no full day of care can be billed

Figure S2: Hospital palliative care and palliative home care use at the end of life in older patients with cancer: percentage of patients with use/contacts in the last 10 days (n=3,546)

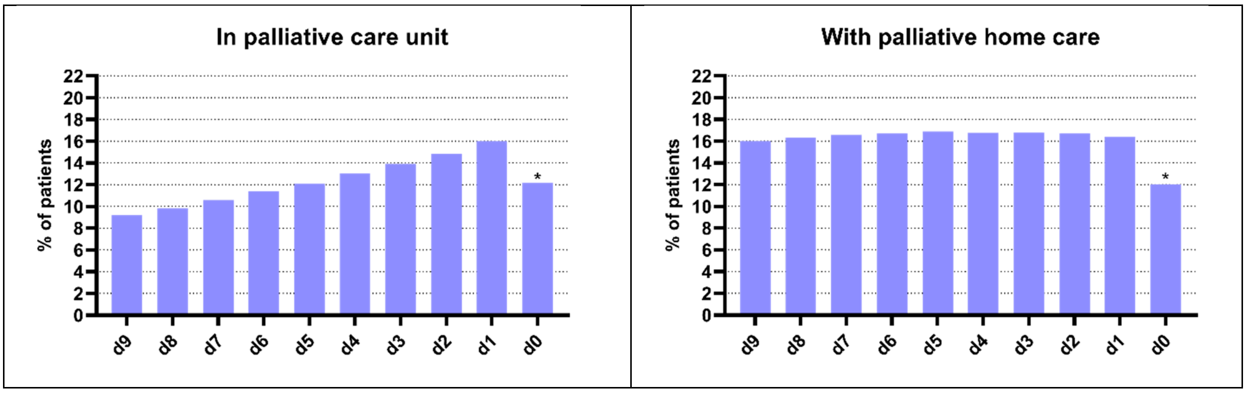

* Because of billing rules, % of patients with use on the day of death might be an underestimation because no full day of care can be billed

Table S6: Sensitivity analysis (baseline nursing home resident status added as co-variate): Logistic regression presenting the association between baseline GS/GA results at new cancer diagnosis and subsequent use of specialized palliative care in the last 3 months of life in older patients with cancer

		Unadjusted analysis		Adjusted analysis [a]	
**Geriatric screening at cancer diagnosis (n=3,546)**					
		OR (95% CI)	P value	OR (95% CI)	P value
G8	Normal vs abnormal	0.93 (0.79 –1.09)	0.3803	0.97 (0.80-1.17)	0.6984
**Geriatric assessment at cancer diagnosis (n=2,761)**					
		OR (95% CI)	P value	OR (95% CI)	P value
Functional status: ADL	Dependent vs independent	0.66 (0.57-0.77)	**<.0001**	0.86 (0.73-1.02)	0.0870
Functional status: IADL [b]	Dependent vs independent	0.64 (0.55-0.75)	**<.0001**	0.80 (0.67-0.95)	**0.0130**
Falls history [b]	Falls vs no falls	0.77 (0.66-0.90)	**0.0011**	0.88 (0.74-1.04)	0.1415
Pain: VAS [b]	Pain vs no pain	1.21 (1.04-1.40)	**0.0156**	1.16 (0.98-1.37)	0.0838
Cognition: MMSE [b]	Mild/severe cognitive impairment vs normal	0.64 (0.53-0.78)	**<.0001**	0.78 (0.62-0.97)	**0.0269**
Depression: GDS-4 [b]	At risk for depression vs not at risk	0.96 (0.82-1.12)	0.6127	0.88 (0.74-1.05)	0.1544
Nutrition: MNA-SF [b]	At risk for malnutrition/ malnourished vs normal	1.39 (1.14-1.70)	**0.0013**	1.05 (0.84-1.32)	0.6492

[a] Adjusted for age, sex, tumor type, stage, Charlson Comorbidity Index, attained educational level, marital status, cohort identification, and time between GA and death + nursing home resident status

[b] Patients with missing data were omitted (number of patients with missing data displayed in Supplementary Table S1)

Footnote: At baseline 4.5% of patients were nursing home residents (n=160, based on IMA data); Bold numbers indicate p-values <0.05

Abbreviations: GS: geriatric screening; GA: geriatric assessment; OR: odds ratio; CI: confidence interval; ADL: Activities of Daily Living; IADL: Instrumental Activities of Daily Living; VAS: Visual Analogue Scale; MMSE: Mini Mental state Examination; GDS-4: 4-item Geriatric Depression Scale; MNA-SF: Mini Nutritional Assessment – Short Form; IMA: InterMutualistic Agency
